# Blockchain Evaluation Approaches: State-of-the-Art and Future Perspective

**DOI:** 10.3390/s20123358

**Published:** 2020-06-13

**Authors:** Sergey Smetanin, Aleksandr Ometov, Mikhail Komarov, Pavel Masek, Yevgeni Koucheryavy

**Affiliations:** 1 National Research University Higher School of Economics, 101000 Moscow, Russia; ssmetanin@hse.ru (S.S.); evgeny.kucheryavy@tuni.fi (Y.K.); 2 Tampere University, FI-33720 Tampere, Finland; aleksandr.ometov@tuni.fi; 3 Brno University of Technology, 61600 Brno, Czech Republic; masekpavel@vutbr.cz

**Keywords:** blockchain, modeling, simulation, emulation, review

## Abstract

The present increase of attention toward blockchain-based systems is currently reaching a tipping point with the corporate focus shifting from exploring the technology potential to creating Distributed Ledger Technology (DLT)-based systems. In light of a significant number of already existing blockchain applications driven by the Internet of Things (IoT) evolution, the developers are still facing a lack of tools and instruments for appropriate and efficient performance evaluation and behavior observation of different blockchain architectures. This paper aims at providing a systematic review of current blockchain evaluation approaches and at identifying the corresponding utilization challenges and limitations. First, we outline the main metrics related to the blockchain evaluation. Second, we propose the blockchain modeling and analysis classification based on the critical literature review. Third, we extend the review with publicly accessible industrial tools. Next, we analyze the selected results for each of the proposed classes and outline the corresponding limitations. Finally, we identify current challenges of the blockchain analysis from the system evaluation perspective, as well as provide future perspectives.

## 1. Introduction

Historically, blockchain systems were designed to support the data immutability among different decentralized nodes [1]. This niche of distributed systems development had already obtained a significant impact on business, touching upon all industries in the world as part of the Distributed Ledger Technology (DLT) paradigm [2]. Today’s blockchain technology applications vary from public means of record-keeping and private storage [3] to connecting various heterogeneous devices as part of the Internet of Things (IoT) paradigm [4]. Moreover, IBM researchers forecast that the DLT market based on blockchain is expected to reach $60.7 billion by 2024 [5], highlighting the timeliness of the systems’ integration needs. The tendency of the blockchain systems’ adoption could be found in almost any digitalized industry, forcing companies to prepare to profit from the blockchain technology integration [6].

Deloitte’s Global Blockchain Survey highlighted that the perception of the blockchain is presently reaching the point of no return, with the corporate focus shifting from exploring the technology potential to creating production business applications [7]. As one of the leading blockchain integration drivers, the financial sector accounted for more than 60% of the global blockchain market value in 2018 [8]. Since then, other industries have been cautious about finding use cases to ensure a good return of investment by implementing blockchain-based applications due to their extreme complexity compared to conventional centralized systems. As a result, more and more entities in more and more spheres, for instance, the biological sciences, media, government agencies, communications, agriculture, and healthcare are broadening and developing their blockchain initiatives to keep up with the pace of the technological evolution [9,10].

Given the enormous interest in blockchain-based solutions, the number of startups is increasing yearly in both private and public sectors [11,12]. From the very beginning of the blockchain era, blockchain-related investments increased from about a million U.S. dollars in 2012 to more than one billion in 2017 [13], currently breaking the line of $23.7 billion [14]. A representative example is the blockchain protocol EOS, which has engendered a significant investment of $4.2 billion in its initial coin placement [15]. While the United States is presently considered a leader in the blockchain segment [16], it is also expected that China will be able to achieve this position in the coming years. One of the central reasons is that China’s investments in blockchain technology are almost doubling each year [17].

A recent survey of business leaders in Europe [16] showed that almost 50% of them expected the blockchain to be added to their current operating business model. Moreover, another 33% claimed they expected the blockchain to be entirely replaced by their current operating model. About 66% of global companies expressed a moderate level of interest in blockchain technology, with almost 10% currently in the process of conducting experiments or implementing blockchain solutions [18].

Cryptocurrencies, and more specifically, Bitcoin, tend to be some of the first and central applications of the broadly known blockchain paradigm, receiving much more attention than other blockchain use cases. As of the third quarter of 2019, Bitcoin reached a high market capitalization of $205.4 billion U.S. dollars [19]. While Bitcoin could be considered the largest, some other cryptocurrencies such as Ethereum, Ripple, and Litecoin have also gained a significant market share in 2019 [20]. Many believe that blockchain and its use in cryptocurrency technology will allow shifting from traditional money transactions to digital ones supported by means of secure ledgers in the coming years.

As a result of the deep blockchain systems’ penetration into our everyday lives, the number of distributed node interactions is expected to increase significantly [21], bringing more load to the energy grid [22], as well as infrastructure and peer-to-peer (P2P) networks and storage volumes [23]. While the field of conventional infrastructure-like (cloud) communication analysis and predictability is already a well-studied topic, the impact of new networking paradigms, such as fog and edge [24,25], is expected to bring a new level of system complexity both from communications and computing perspectives. Therefore, blockchains provide some unique differences from everything that has come before. A blockchain survives faults and attacks by the use of redundant checking of multiple nodes. This resiliency goes far beyond replication since it happens across the network without any central coordinator or intermediary [26]. Generally, the design of said distributed systems requires careful planning and performance evaluation, while conventional approaches may face numerous challenges due to the increase in the complexity.

To date, there is still a lack of unified tools and instruments for the performance evaluation and behavior observation of blockchains, while the number of related applications is already sky high [27]. Blind development of blockchain-based extraordinarily complex and dynamic systems without any preliminary performance evaluation may have a tremendous negative impact during the actual deployment phase. In most cases, current evaluation approaches are based on the emulation techniques that imitate and replicate the behavior of the entire network. Evidently, it requires a significant amount of computational, storage, and communications resources [28,29]. Little attention is also given to deploying real testnets driven by the community, but it requires significant incentivization activities to get the users involved [30,31]. Consequently, the emulation entails a massive problem of scalability for real-world deployments’ evaluation [32]. Moreover, such an evaluation requires considerable engineering efforts to modify complicated open-source solutions or production systems to test out continually evolving systems in a timely manner. In this case, modeling approaches, for instance, analytical and simulation methods, can be considered as an alternative trading precision for the evaluation speed.

The main research question highlighted in this paper is:


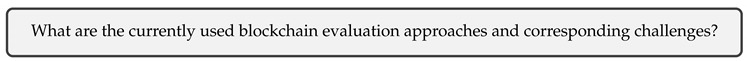


This paper also aims at future prospects of blockchain simulation/modeling approaches, as well as at surveying existing solutions utilized for blockchain systems’ performance evaluation. It aims to provide an overview of existing strategies found in the literature, as well as in open access sources in order to provide the linkage between the approach and solved task or its general area.

The rest of the paper is organized as follows. The next section provides the applied critical review methodology. Section 3 highlights the main background information, as well as the motivation of this paper. Section 4 outlines the examples of the most widely used approaches applicable to blockchain evaluation and related classification. Next, Section 5 covers the main analytical and simulation tools used for blockchain evaluation. Section 6 outlines the main emulation-based approaches used by industry and integrators. Next, Section 7 lists the main challenges related to the blockchain evaluation from both execution and legal perspectives. It also provides recommendations on the future improvement of the modeling process. The last section concludes the paper.

## 2. Methodology

In order to identify the key publications on the evaluation of blockchain technology, we performed a literature search in scientific databases following PRISMA guidelines [33] with minor modifications. The analysis covered leading computer science journals and conferences: IEEE Xplore, ACM Digital Library, ScienceDirect, SAGE Journals Online, Springer Link, etc. To find relevant articles and papers for our research, we applied the following search string: (Blockchain OR “Distributed Ledger”) AND (Simulation OR Model OR Modeling OR Emulation OR Evaluation).

In total, we gathered a set of 1432 potentially relevant publications, excluding grey literature and pre-prints. We removed potential duplicates and arrived at 960 resources. We then analyzed the titles, keywords, and abstracts of the publications to identify papers and articles that described at least one modeling or simulation approach for blockchain-based systems. In doing so, we selected a total of 44 publications. To further extend our literature sample, we analyzed the references of the selected publications for additional papers or articles relevant to our research. Following this process resulted in a total of 63 publications. Finally, we analyzed publicly-available solutions proposed by the actual blockchain developers and arrived at a total of 71 publications.

Once the literature selection process was completed, we carefully read the selected sources to identify the described strategies and challenges. Next, we classified the extracted approaches into five general groups.

## 3. Background and Motivation

This section elaborates on the identified approaches presently used for the blockchain evaluation from analytical, simulation, and emulation perspectives. The majority of modern software systems rely on numerous practical and theoretical approaches for their performance evaluation. Nonetheless, Software Development Life Cycle Models (SDLC) fall short in providing the necessary flexibility for describing blockchain systems still keeping the priority of the initial performance evaluation before the actual system deployment, as well as followed by continuous reevaluation and testing [34]. Since the operation of the highly dynamic environment showed itself to be unstable multiple times in the past, the same should be applied to any pre-deployment phase of the blockchain systems.

Today, simulations and analytical modeling are standard instruments for the behavior and performance evaluation of the majority of blockchain-based solutions [35]. At the same time, analytical modeling could also be applied to the blockchain evaluation for cases when a mathematical model has a closed-form solution, i.e., a simplified description of the system operation should be given in the form of a “set of equations” utilized to describe the behavior of the system formulated as a mathematical analytic function.

Simulation models could be either a pure but simplified simulation of the system behavior or, more often, could be considered as a subclass of mathematical models. In this case, the simulation would combine both mathematical and logical aspects of the system and try to replicate a real-life system behavior using computer software. Simulation tends to be used in cases when the analytical description cannot be formulated, or creating an analytical model is fundamentally impossible. Simulation attempts to approximate a system’s behavior and development over time by running a model [36]. Those models are, in essence, generally simplified abstractions of a simulated system that aim at covering the specific level of details required to achieve the research goals. Simulations can demonstrate some limited effects of alternative conditions and action paths. This group of evaluation approaches is also used in cases where a real system cannot be engaged, for instance, when the system is not accessible, or it may be dangerous or unacceptable to access the system, or the system does not exist [37].

More broadly, computer simulation tries to approximate the system behavior and development over time by implementing and running a computer simulation model. By changing variables and conditions in the implemented simulation model, researchers can make predictions about the behavior of the simulated system without the need for the actual implementation of the entire system. A computer simulation is commonly used when it is a complicated task to accomplish the system emulation and for emulating the blockchain system as part of more complex environments [38].

According to [39], the simulation models can be classified along different dimensions: continuous or discrete, static or dynamic, deterministic or stochastic. A discrete model is a simulation model in which the state variables change only at a discrete set of points in time. A continuous model is a simulation model in which the state variables change continuously over time. A minimal amount of real-world systems is wholly continuous or discrete, but in the majority of cases, one type of change predominates over the other. A static system simulation model, which is also called Monte Carlo simulation, represents a system at a given point in time or represents one in which time does not matter. As the opposite of a static model, a dynamic simulation model represents a system with varying behavior over time. A model is called deterministic in the case that it does not include any random or probabilistic variables. Otherwise, it is called a stochastic simulation model.

Besides analysis and simulations, we can consider a standalone group of approaches used for the blockchain performance evaluation: the emulation of the entire or part of the system. It is the process of imitating the behavior that can be observed from the outside to match an existing target. The emulation mechanism’s internal state does not need to reflect the internal state of the emulated system accurately. On the other hand, simulation involves modeling the internal state of the analyzed system, i.e., the output of a good simulation is that the simulation model will emulate the simulated system. Emulators, therefore, require significant overhead to run the emulated interface and/or system’s functionality in real-time while providing the layers of virtualization needed to emulate a whole system. As a consequence, emulation seems to be more accurate in comparison with simulation but requires much computational resources to achieve it at the same time.

**Figure 1:**
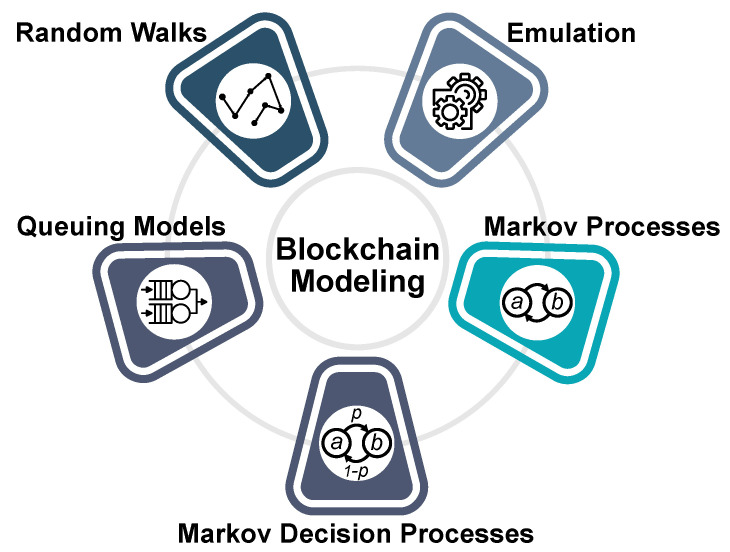
Proposed blockchain modeling approaches’ classification.

Currently, the field of basic modeling theory of blockchain systems is still in its infancy, including but not limited to constructing mathematical models, providing security and performance analysis, and identifying potential optimization points. In order to identify the publications primarily addressing blockchain modeling, we performed a search on a range of scientific databases that cover the high-quality computer science journals and conferences. Based on the literature review, we divided blockchain evaluation approaches into the following classes (see Figure 1): queuing models, Markov processes, Markov decision processes, random walks, and emulations. Some systems may have several types of models so that we can assume such a system as a mixed approach. The proposed classification is an extended one from [40]. The following subsections provide a more detailed overview of each technique.

## 4. Evaluation Strategies and Metrics

In order to better understand the reasons behind the need for blockchain evaluation, this section outlines the main metrics of interest and evaluation strategies applied in this process. To date, the academic literature lacks explicit analysis of the strategies due to the nascent field of research.

Generally, any blockchain-based system could be analyzed from various perspectives: usability, block analysis, functional testing, security analysis, integration, smart contracts, DApps, networks, and especially, performance evaluation, which is the main focus of this paper. Performance testing allows us to analyze the network size, and its ability to process transactions is critical, as it provides an opportunity to identify hardware and software bottlenecks in advance in addition to future Operational Expenses (OPEX). Performance-wise, the operation of a blockchain could be described by the set of the main metrics that could be classified into three main groups, as summarized in Table 1 (some metrics have multiple terms since the taxonomy is not yet defined by the community.): Blockchain metrics (the number of produced blocks, the number of processed transactions, processing time, finality time, etc.), P2P network metrics (the number of hit/miss requests, the number of active peers, the volume and structure of P2P traffic, etc.), and system node metrics (CPU, memory, storage, network, etc.).

Most of the metrics have the lower/average/upper bounds defined by the system configuration, network, or hardware limitations. Thus, developers should carefully consider those during the system design phase, while the numbers usually found in the project descriptions may be somewhat speculative, e.g., tps could not be measured in an objective way since it will require analysis on all the blockchain nodes, which is mostly unfeasible in a real-life environment. Similar problems could be found at lower levels, i.e., being related to the distributed P2P network operation. The heterogeneity of the communication environment, as well as unpredictable node distribution, may have an extensive impact on the metrics of interest, including block delivery and transaction validation times. Notable for enterprise (mainly private) blockchains is the specific definition of system metrics, e.g., a developer might measure transaction latency on public blockchains as when a transaction is available on 80% of nodes. However, for widely used enterprise consensus mechanisms, the threshold will often be set to 100%.

In truth, blockchain developers should consider the following metrics during the system design since the adjustments of already deployed distributed ones may be very challenging or even impossible.

## 5. Analytical and Simulation-Based Approaches

This section outlines the main academic activities related to the blockchain evaluation, while the industrial ones are given in Section 6. The summary of both groups is given in Table 2.

### 5.1. Queuing Models

A Queuing Model (QM) is a mathematical model allowing the prediction of the queue lengths and waiting time in the system. Some preliminary work on QM for a blockchain was carried out several years ago by Kasahara and Kawahara [41]. This work studied the applications of queuing theory in the context of transaction-confirmation time for Bitcoin. The authors investigated and described the relationship between the demand for transactions with low fees and the transaction-confirmation time. Additionally, the authors demonstrated that the enhancement of the maximum block size appeared as an inefficient way to reduce the time of transaction confirmation. The development of a queuing analytical approach to the blockchain was continued in the work [42]. In both papers, the authors made the assumption that the transaction confirmation time follows a continuous probability distribution function. In order to define a system of differential-difference equations, the authors utilized the supplementary variable method by using the elapsed service time. Unfortunately, the authors did not provide the correctly defined solution of the formulated system. To overcome this issue, Li et al. introduced a generalized queuing theory of blockchain systems through the matrix-geometric solution in [43]. The proposed theory utilized different exponential service stages for times of blockchain-construction and block-generation.

Later on, Memon’s group developed a new simulation model of the mining process for blockchain systems using queuing theory in [44]. The research group from Yale University proposed a stochastic model, which aimed at capturing the blockchain network dynamics and evolution [45]. Ricci et al. proposed a complicated framework composed of the machine learning model and queuing theory model [46]. It aimed at solving two significant tasks: identifying which transactions will be confirmed and characterizing the confirmation time of transactions.

Several recent studies utilized the fluid limit: a subsection in queuing theory describing deterministic processes, which aims at an approximation of the evolution of the analyzed stochastic process. For instance, the research group from the University of Amsterdam developed a Bitcoin-inspired infinite-server model with a random fluid limit [47]. Moreover, King et al. considered the fluid limit of a random graph model for a shared ledger and distributed ledger in blockchain systems [48].

### 5.2. Markov Processes

A Markov Process (MP) is a basic mathematical tool for performance evaluation of blockchain systems proposed more than 10 years ago in [49]. It describes a process by which future probabilities are determined by the most recent values. A stochastic process can be called an MP only in the case if:

(1) $$P\left(X_{t_{n}}⪇X_{n}|X_{t_{n-1}},\cdots ,X_{t_{1}}\right)=P\left(X_{t_{n}}⪇X_{n}|Xt_{n-1}\right)$$

is true for every *n* and every $t_{1}<t_{2}<\cdots <t_{n}$ [50]. From the mathematical point of view, an MP is a first-order autoregressive model $X_{t}=c+aX_{t-1}+\epsilon_{t}$, where $X_{t}$ is a time series, *a* is a parameter of the model, *c* is a constant, and $\epsilon_{t}$ is a white noise.

Some preliminary work was carried out by Eyal and Sirer in [51]. The authors constructed a basic Markov process for analyzing the vulnerability of blockchain protocols. They found out that selfish mining tends to become profitable in case the hashing power of a miner is larger than 25%. This approach was further developed by Nayak’s research group by expanding the mining strategy with a novel “stubborn” strategy [52]. According to the results of the systematic exploration of strategy space, researchers found that the revenue of the attacker tends to increase by non-trivial combinations of network-level attacks and stubborn mining. In the paper [53], the authors explored the relationship between the existence of multiple misbehaving pools and the profitability of successful selfish mining.

The research group from Northeastern University developed a simple MP-based approach for analyzing the consistency properties of blockchain protocols [54]. While previous studies on consistency [55,56] argued that Markov models are too complicated for analysis, this research was the first one successfully using the MP-based models to analyze consistency against any adversary.

### 5.3. Markov Decision Processes

A Markov Decision Process (MDP) is a discrete time stochastic process, which is widely used as a mathematical framework for a sequential decision-making task with a fully observable environment described by a Markov transition model and additional rewards. MDP can be defined as a four-tuple $\left(S,A,P_{a},R_{a}\right)$, where *S* is the finite set of states, *A* is the finite set of actions, $P_{a}\left(s,s^{\prime }\right)=Probability\left(s_{t+1}=a|s_{t}=s,a_{t}=a\right)$ is the probability that action *a* in state *s* moves to state $s^{\prime }$ at state $s_{t+1}$, and $R_{a}\left(s,s^{\prime }\right)$ is the expected reward received immediately after transitioning from state *s* to state $s^{\prime }$ by performing action *a*.

In order to identify an optimal mining policy in blockchain systems, several studies utilized MDP as a mathematical modeling framework. For instance, several studies [57,58,59] utilized MDP to find the optimal selfish mining strategy. Bitcoin Simulator [58] was initially implemented to examine how network characteristics, modification of the consensus protocol, and consequence parameters affect the efficiency, security, and scalability of Proof-of-Work (PoW) powered blockchain systems and is also partially based on MDP. The framework consists of two main components: a blockchain simulator and a blockchain security model utilizing the MDP approach. In order to make the simulator as realistic as possible, the authors incorporated real blockchain networks’ statistics, for instance, the number of nodes, the geographical distribution of nodes, block size distribution, time of block generation, network delays, and techniques for information propagation. The primary output of the blockchain simulator component is the stale block rate, which is then processed by the blockchain security model. The output of the security model, which is based on MDP, enables researchers to identify optimal adversarial strategies by comparing the performance and security of blockchain systems with different parameters. Based on the comparison of the security aspects of Bitcoin and Ethereum, the authors found that in order to match Bitcoin’s security with six block confirmations, Ethereum needed at least 37 block confirmations. Bitcoin Simulator is implemented based on the widely-known discrete-event Network Simulator 3 [60], which has a scalability limit of 6000 nodes. Bitcoin networks currently have more than 10,000 nodes as of May 2020 [61], so technically, Bitcoin Simulator is not able to perform the simulation of the current state of the entire Bitcoin network [62].

### 5.4. Random Walks

A Random Walk (RW) is a stochastic mathematical model that describes a path that consists of random changes or steps on mathematical space at discrete points in time, widely utilized for various Information and Communications Technology (ICT) systems’ analysis [63,64]. Given independent and identically distributed random variables $X_{1},X_{2},\cdots ,X_{n}$, where $X_{i}\in R_{n}$, the pure structure of random walks can be defined as $Yn=\sum_{1}^{n}X_{i}$.

The authors of [65] refined Nakamoto’s model [23] to study the double-spending attack issue in blockchains, primarily focusing on the likelihood of attack success. Jang and Lee [66] proposed a new model, which takes into account block confirmation release in contrast with Goffard’s model. Grunspan and Perez-Marco, within their research [66,67], computed the minimal number of confirmations to be requested by the recipient such that the double-spend strategy was non-profitable.

To summarize, most of the blockchain evaluation approaches based on analytical modeling and simulations are generally simplified abstractions of the actual systems aiming at a specific focus on particular aspects of the system operation, thus providing highly limited observations on the main blockchain evaluation metrics listed in Table 1. Therefore, those are generally found in academic works in contrast to another broad field of the system emulation driven by actual integrators.

## 6. Emulation-Based Approaches

Emulation (E) is the process of imitating the behavior that can be observed from the outside to match the selected target, which is mainly used by the actual blockchain developers having access to the actual system code. Most of the industry and integrators apply this approach to test the performance of their private or consortium blockchain-based solutions, while public ones obtain much less attention. The details on the main blockchain emulation examples are summarized in Table 3. The reasoning behind this is that the first two groups originate from highly directional business processes driven by corporations that follow conventional SDLC strategies in order to decrease CAPEX and OPEX costs. Generally, blockchain emulators are expected to provide an overview of the actual system operation, therefore providing a more in-depth analysis of the system metrics from Table 1.

To start with, an industrial project titled Hyperledger [68] is one of the blockchain-adoption drivers known to the world today. Started in 2015 and driven by both the community as well as Linux Foundation, it has already received contributions from IBM, Intel, and SAP Ariba. The project has resulted in a set of DLT tools, including Hyperledger Composer and Caliper, aiming to support Hyperledger Fabric pre-deployment activities, unfortunately lacking many features of the actual blockchain operation [69]. The main idea behind this tool development is to allow easy and on-the-fly testing of smart contractsin a variety of simplified system operation abstractions, thus accelerating the actual deployment process. It consists of a data model, a set of transactions, and a set of queries by which those transactions can access data within the model, thus allowing for the execution of various microservices in the emulator. A number of academic activities were presented with respect to this framework [70].

Another emulation tool is Blockbench [28], which is an academia-driven framework for stress testing and analyzing private blockchains. It measures component-wise and overall system performance in terms of scalability, throughput, latency, and fault-tolerance. The authors conducted experiments on Hyperledger Fabric, Parity, and Ethereum, showing that these blockchains were still far from replacing current database systems in the case of traditional data processing workloads. The authors claimed that any private blockchains could be emulated or used nodes could be instantiated in the network.

Next, VIBES [71], a public blockchain emulator, was initially designed to correct the deficiencies in the currently stopped Bitcoin-Simulator project [72]. In order to achieve this goal, VIBES upgrades the scalability characteristics of the Bitcoin-Simulator by spanning multiple threads to carry out expensive computations and achieve full CPU utilization. As Bitcoin Simulator, VIBES utilizes the same method of inserting timestamps to execute the PoW hypothetically. Both simulators appear to be trailblazers to simulate the entire blockchain network. Moreover, in contrast with previous simulators, VIBES is designed to simulate transactions in the blockchain network. As an input, VIBES processes the network parameters, as well as the blockchain system-wide characteristics. Previous input parameters joined with theoretical and empirical results are used to simulate the blockchain system with a vast number of nodes. Based on these data, VIBES does not require performing heavy computations so that it can speed up the simulation process. However, the system scalability in the experiments is kept below 1500 nodes.

BlockLite [62] is another tool designed to emulate the public blockchain operation on a single node with both high usability and scalability. In contrast to VIBES, BlockLite is comprised of a module to execute real PoW workload, scales up to 20,000 nodes, and operates as a fully decentralized system. In order to deal with the scalability challenge of the real PoW loads, BlockLite delegates one node to solve a puzzle in a pre-running stage and then replicates the behavior of the delegation node in a running application.

A particular niche of blockchain evaluation is related to private blockchains. Here, the developer may either develop his/her completely proprietary blockchain solution or base it on one of the open systems adopted for private use. One of the examples of the last group is Ethereum [73]. Many developers base their private blockchain systems on Ethereum, varying from conventional finance to industrial applications [74], by means of an enterprise solution. Customer-oriented projects require initial performance evaluation, and thus, Ethereum Tester was specifically designed for the deep emulation of said systems [75]. Besides observing the specific metrics stated, Ethereum Tester allows analyzing the operation of the Application Programming Interface (API), web integration, back-end, smart contracts, and several other blockchain tests.

Another toolbox utilized for private Ethereum-based blockchains’ evaluation is known as Truffle Suite [76]. It is composed of a set of tools, including blockchain emulation, smart contacts, and transaction tracing in a virtual machine. The system is composed of three main components covering the main operational aspects [77]. The first component, called Truffle, is the actual development environment that integrates the compilation, testing, and deployment of smart contracts. Ganache is the second one. It is a locally deployed blockchain simulator featuring a graphical user interface that can simulate blockchain networks and live-test smart contracts without requiring setting up real test networks or using a remote network. The last one is Drizzle, being an assortment of front-end libraries that offer useful components for developing web applications that can seamlessly connect with the smart contracts. The system is extremely flexible, but dedicated only to Ethereum analysis.

A massive open-source environment called Corda Testing Tools provides a broad range of functionality, including unit tests and integration tests for both small projects and enterprises from the transaction perspective designed explicitly for R3 Corda project analysis [78]. The main idea behind the project is to analyze the potential of interoperability between different parties with personal blockchain systems. The performance of Corda significantly varies based on the hardware and system complexity, e.g., from 8 to 1001 tps according to the official documentation [79].

One more toolbox for private blockchain integration testing is Exonum Testkit [80]. Its main targets are testing the logic of transactions and analysis of custom APIs in the Exonum blockchain, as well as the lack of consensus algorithms’ evaluation. Exonum Testkit is written in the Rust programming language, but also provides a Java Binding tool as a part of its software development kit (SDK).

It must be noted that there are some minor projects developed to analyze a particular part of the blockchain operation, but not for the actual blockchain evaluation, thus falling out of the scope of this paper. For example, Manticore [81] is a tool developed to analyze the behavior of Ethereum smart contracts from the dynamic symbolic execution perspective [82]. This field may be of interest for virtual environment developers. A similar analysis at the low level was done in [83,84]. Some other projects are dedicated to the Distributed Application (DApp) deployment strategies in Ethereum as Truffle Drizzle [85]. Numerous other tools are dedicated to the standalone node emulation in Ethereum, such as Geth [86] or EthereumJS Monorepo [87]. BitcoinJ is a similar project, but utilized for Bitcoin. It is a Java-based implementation of the Bitcoin protocol for the emulation of transactions and wallet operation [88]. It has a number of abstractions allowing users to analyze the simplified payment verification, asynchronicity, and per-connection status. The main drawback of those is the limitation in evaluating the overall system operation.

To summarize, one of the critical aspects of constructing emulation models tends for the academic segment to be the computational resource limitations. Broadly utilized solutions for this issue are to simplify resource-competitive modules, utilize multi-threading programming in model implementation, and to deploy emulation models in the cloud. The impact of resource limitations for product developers and integrators is less critical; thus, most of their activities are targeted at the emulation environment. It allows users to obtain more detailed information on the system operation. Evidently, a unified solution for blockchain emulation does not exist, while this section listed the main aspects of existing projects in this field, providing the reader with an opportunity to select the tool of interest depending on the application from Table 3.

During the review, we identified that major analytical models are based on Markov processes, which are commonly utilized for examining the incentive-compatible property of blockchain systems, studying selfish mining attack strategies, and evaluating the consistency properties of blockchain protocols. At the same time, models based on Markov decision process are commonly used for obtaining relatively better results on examining selfish mining attack strategies. The majority of papers aiming at researching double spending attacks in blockchain systems are based on the random walks approach. Emulation approaches should be applied in cases when the detailed performance evaluation is required.

## 7. Current Challenges and Future Prospects

**Figure 2:**
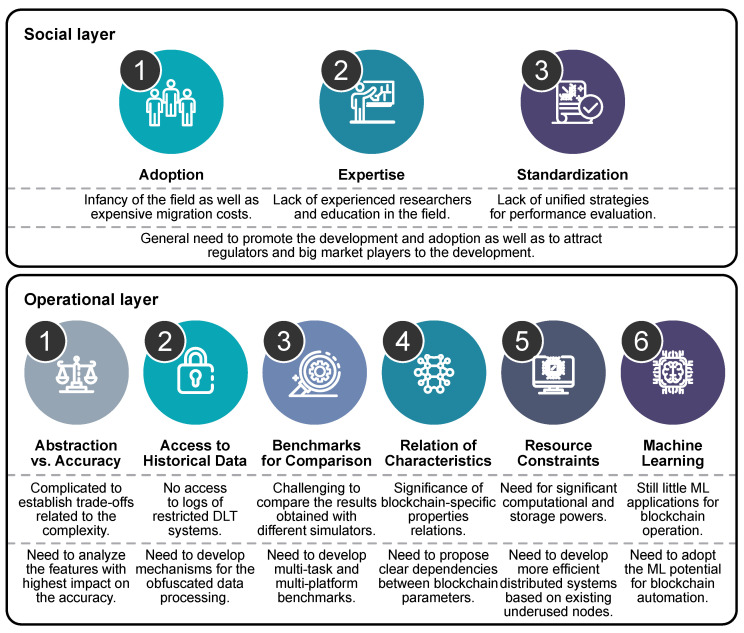
Challenges and perspectives of blockchain simulation systems.

The models listed in Section 3 primarily focus on merely imitating and replicating the behavior of blockchain networks, while reducing the number of computational resources in contrast with the original blockchain implementations. Based on the analyzed literature, we identified two groups of challenges and prospects explicitly related to blockchain evaluation rather than to general blockchain operation; see Figure 2.

### 7.1. Lack of Adoption

The first and the main social challenge is related to limited adoption of the technology. Adding to developers’ bad luck, many affiliate blockchains with cryptocurrency used for money laundering and tax fraud [89]. It has not publicly received much support from the government due to its historical opposition to control [90]. Besides, the mass adoption of the technology remains very limited due to numerous factors, e.g., institutional, environmental, technological and many others [91]. In contrast, the top management of companies already shows interest in deploying blockchain-based systems, according to [92], which is expected to result in additional activities related to the developed systems’ evaluation needs.

### 7.2. Lack of Expertise

One of the main challenges of today’s blockchain system developers is a lack of skills or experience in analyzing and developing blockchain applications. The steps from centralized to massively adopted distributed systems are only to be made in the coming years. Learning additional skills or understanding the best practices to implement blockchain applications is costly. It is essential to manifest the fact that blockchain technology has enormous potential for simplifying all the bureaucratic procedures for the state and making transactions transparent and more accessible, thus pushing the need for the systems’ evaluation to a higher priority.

### 7.3. Lack of Standardization

Based on the literature review, we can state that the evaluation of blockchain systems highly varies depending on the type, i.e., private, public, or consortium [93]. Generally, public blockchains are analyzed by targeting some specific research goals, while private and consortium blockchains are driven by business needs (reducing CAPEX/OPEX, system planning, peak performance evaluation, etc.). This is one of the results due to which most of the high precision evaluation tools are provided by private blockchain developers and lie in the emulation class. Moreover, the audit or analysis of private blockchain systems may sometimes be even impossible due to internal company regulations or the General Data Protection Regulation (GDPR); it may be beneficial to define a common standardized approach for private blockchain evaluation in order to reach consistency while comparing different enterprise products.

### 7.4. A Multi-Task Benchmark for the Models’ Comparison

Even if several articles focused on one problem [52,53,57,58,59], it may be challenging to compare the outcomes in a straightforward way since even a slight difference in the test data may affect the final results significantly. Thus, one of the significant current challenges can be formulated as the lack of a multi-task benchmark and analysis platform for performance and accuracy evaluation of simulated models. In order to overcome this issue, new experimental studies should rely on the experience of developing such a multi-task benchmark and analysis platform for other computer science fields of study. For instance, in computational linguistics, GLUE [94] was developed for analyzing and evaluating the performance metrics of models across a wide variety of existing natural language understanding tasks.

### 7.5. Access to the Representative Historical Data of Blockchain Systems

Following the previous challenge, historical data such as logs collected from monitoring nodes or general network statistics are commonly analyzed and utilized in simulation models. However, this approach suffers from several drawbacks. For instance, in the case of obtaining data from specific nodes, these data are under the analyzed blockchain system factors. As a consequence, it may not be representative to examine the blockchain system, which is under other network factors. Besides, these data are observed from logs of a limited amount of nodes, so they are not able to describe other nodes’ behavior [95]. Moreover, in both cases, it can be a challenging task to collect these data from private networks. Blockchain developers and blockchain system owners can solve this challenge by publishing representative statistics from different levels of blockchain networks.

### 7.6. Abstractions Used in a Simulation Model May Affect the Accuracy of the Model

Event-based simulation models, e.g., [71,96], perform the simulation by abstracting part of the node logic of the whole node into a range of discrete events triggered at scheduled periods of time. Even if these abstractions increase scalability and cost-effectiveness, they may ultimately result in the exclusion of the essential traits of blockchain systems due to the abstraction of the nodes’ functionalities [95]. One of the possible, but rather complicated solutions is to validate empirically the influence of abstracted functionalities on the blockchain system by conducting comparison tests.

### 7.7. Relations Between Blockchain Characteristics

While certain relations or even trade-offs between quantitative blockchain characteristics were deeply examined, e.g., the effect of the block-size limit to the transaction-confirmation time [41,42], the relations between more complicated characteristics have remained open. For instance, several blockchain characteristics are challenging to assess, e.g., cost, law, and regulation. As a consequence, in order to simulate systems that take into account these characteristics, more detailed and testable criteria must be generated. Some outstanding work in identifying trade-offs between distributed ledger technology characteristics was conducted in the paper [97], so it can be used as a starting point for further analysis of this field.

### 7.8. Resource Constraints

In the case of modeling blockchain systems, running a simulation or an emulation may require significant computational resources in order to achieve relevant and accurate results. The basic approaches for reducing the need for computational power are to simplify resource-consumptive modules or procedures in the model implementation, or to utilize a certain central processing unit’s abilities such as multithreading, or to exclude some modules from the model. More advanced and combined approaches may be required to run the execution of a large-scale blockchain model on tens of thousands of nodes in the cloud [62].

### 7.9. Machine Learning in Simulation Modeling

While Machine Learning (ML) solutions demonstrated an ability to perform successfully in system modeling tasks [98,99,100,101], a minimal amount of studies applied those for the modeling of blockchain-based systems. For instance, the Markov chain neural networks concept introduced by Awiszus and Rosenhahn [102] has great potential in the application of Markov chains for blockchain modeling. At the same time, the Markov Decision Process Extraction Network (MPEN) [103] can potentially be utilized in order to extract automatically minimal relevant aspects of the dynamics from observations to model a Markov decision process.

Based on the above, we can conclude that there exists a number of challenges to be addressed by the research and professional community pushed by the growing industrial and academic interest in blockchain modeling. Several issues, e.g., the development of a benchmark, the estimation of the influence of model abstractions, and the relations between blockchain characteristics, can be solved by researchers on their own, whereas some other challenges, e.g., access to representative historical data, could be overcome only by the whole blockchain-development community participating. At the same time, a few potential directions, such as machine learning techniques and cloud-based deployment, seem to be able to enforce the simulation accuracy and performance notably.

## 8. Conclusions

In this paper, we executed a critical review, analyzed the state-of-the-art of blockchain evaluation approaches, and identified current challenges and future prospects of blockchain simulation and modeling.

Firstly, we outlined the main motivation and background. Next, we listed the main perspectives and metrics that could be evaluated. Further on, we proposed a classification of blockchain modeling approaches into the following classes: Queuing Models, Markov Processes, Markov Decision processes, random walks, and emulations. We executed the literature review based on the PRISMA methodology extended by the industrial projects and reviewed selected papers for each of the proposed classes. Finally, we outlined current challenges and future perspectives in the area of blockchain evaluation.

Based on the critical review, we concluded that analytical and simulation approaches based on queuing theory are mainly utilized for evaluating different blockchain architectures before deploying them over the blockchain network and designing the fluid limit of a random graph model for a shared ledger. Approaches based on Markov processes are commonly used for evaluating the consistency properties of blockchain protocols and studying various attack strategies. As an extension of the Markov processes, approaches based on Markov decision processes are commonly applied for obtaining relatively better results on examining selfish mining attacks. Approaches based on random walks are primarily used to examine double spending attacks in blockchain systems. Emulation is used when the computational and storage resources are not critical in contrast to previous ones. Those are mainly applied by actual developers and integrators when detailed system analysis is required for planning of private and enterprise blockchains.

Finally, we identified current challenges and future prospects of blockchain simulation approaches, which include a lack of expertise, the need for developing a multi-task benchmark for reliable models’ comparison, gaining access to the representative historical data of blockchain systems, evaluating the influence of abstractions on the model accuracy, exploring relations between blockchain characteristics, reducing computational resources for simulation, and the usage of machine learning for better performance. We foresee that one of the main bottlenecks of blockchain evaluation adoption is the need for standardization activities, which is expected to be resolved in the oncoming years. 

## Figures and Tables

**Table 1:** Main blockchain performance evaluation parameters (P) and metrics (M).

	Metric	Type	Description	Main Challenge
	Consensus	P	The distributed process by which a network of nodes provides a guaranteed unique order of transactions and validates the block of transactions.	Inappropriate selection of a consensus algorithm may have a tremendous negative impact on the node and system operation.
	Transaction throughput	M	The rate at which valid transactions are committed by the blockchain network in a defined time period, commonly transactions per second (tps).	Careful selection of the system parameters is required in order to achieve the desired tps.
	Transaction type/size	P	The amount of data in the transaction to be added in the next block.	Inappropriate selection may have increased transaction fees in public blockchains.
Blockchain metrics	Block size	P	Defined size of the block, essentially the number of transactions to be included. It could be used to control the blockchain operation.	Larger blocks may have a negative impact on OPEX and decrease the tps.
	Chain size	M	The total size of the blockchain minus database indexes in megabytes.	A chain that is too long significantly decreases its distribution time to allow the new node to start the operation.
	Network-wide latency (commit time or transactional Latency)	M	The amount of time taken for a transaction to take effect to be used across the network.	Commit parameters may vary in different systems, e.g., 90% in public blockchains or 100% in enterprise private blockchains.
	Finality time	M	The moment when a transaction is committed and can no longer be reversed, i.e., the data cannot be rolled back to the previous state.	Defined in a consensus algorithm, and a threshold should be carefully selected during the evaluation. Otherwise, the finality time should significantly decrease the system efficiency.
	Network size (number of active peers)	M/P	The number of validating nodes participating in consensus excluding observer nodes.	n/a
Network metrics	Volume of P2P traffic	M	The amount of cumulative traffic generated by active nodes in the system.	Operation over public networks may have a tremendous negative impact on energy consumption, connection quality, as well as increased OPEX.
	Structure of underlying traffic	P	The structure of blockchain-related packets (data, service, etc.).	Inefficient selection may cause unnecessary traffic overheads.
	Packet loss ratio	M	The ratio between lost and sent packets related to blockchain operation.	Increased packet loss may increase delays and decrease tps.
	CPU/GPU	M	Hardware utilized for blockchain-related data processing.	Has a significant impact on the involvement in the blockchain operation, as well as on the Capital Expenditures (CAPEX) and OPEX.
	Memory	M	The amount of RAM required for efficient transaction/block processing.		
	Storage volume	M	Local storage space required for the blockchain.		
Node metrics	Connectivity	M	Various metrics of the selected communications technology, its channel quality, reliability, latency, etc.		
	Read latency	M	The time between the read request submission until the reply is received.	n/a
	Read throughput	M	A measure of how many read operations are completed in a defined time period expressed as reads per second (rps).	n/a
	Cache Hit Ratio (CHR)	M	Measurement of how many content requests a cache is able to fill successfully, compared to how many requests it receives.	Low CHR, mainly caused by hardware, may have an impact on the operational speed of a specific node.

**Table 2:** State-of-the-art of the blockchain evaluation analytical and simulation approaches.

#	Field of Study	Str.	Specifics
[41,42]	*Bitcoin:* Transaction-confirmation time	QM	The authors made the assumption that the times of the transaction confirmation follow a continuous probability distribution function.
[43]	*Bitcoin:* The queuing theory of blockchain systems under a dynamic behavior setting.	QM	Analysis of the average mean of transactions in the Memory Pool (MemPool), mean number of transactions in a block, and mean transaction-confirmation time. It is noted that considering more general blockchain queuing systems is required in the future.
[44]	*Bitcoin:* Behavior of blockchain-based applications before deploying them over the blockchain network	QM	The authors assumed that the number of arrivals to the system was a little more than those serviced at the mining station; however, in Bitcoin systems, the number of accumulated unconfirmed transactions can be observed to increase throughout the day.
[45]	*Ethereum:* valuation of selfish mining strategies’ impact.	QM	The work was focused on measurements, blockchain design, and analysis. It was stated that careful examination of the various system parameters’ relationships and related trade-offs should be a base for similar models’ development.
[46]	*Bitcoin:* Profitability of double spending attacks and delays.	QM	The authors assumed no off-chain payment mechanism with no simplified payment verification during the transaction confirmation. They stated that QMs were the most suitable for the analysis of transaction delays.
[47]	*Bitcoin:* The fluid limit of a random graph model for a shared ledger.	QM	It was stated that a more realistic FIFO-batch departure discipline is required in order to analyze a natural G/M/∞-like Bitcoin queue variant of the G/M/∞-like Bitcoin.
[48]	*IOTA:* The fluid limit of a random graph model for a shared ledger.	QM	The author attempted to analyze IOTA scholastically and find the differential equation for the delays in the system, i.e., a converged fluid limit.
[51]	*Bitcoin:* Incentive-compatible property of Blockchain systems.	MP	The authors highlighted the importance of the chain-quality property since if the adversary controls a suitably limited amount of hashing power, then the adversary is also limited in terms of the number of blocks it has added.
[52]	*Bitcoin:* Selfish mining attack.	MP	The authors stressed that a malicious user in this system would eventually succeed in case he/she executed double-spending simultaneously, thus defining a new research direction of stubborn mining.
[53]	*Bitcoin:* Selfish mining attack.	MP	The work observed the relation of the number of malicious selfish miners vs. the selected one success rate.
[54]	*GHOST(Ethereum):* Consistency properties of blockchain protocols.	MP	The work proposed a model that considered various attacks and aspects of the protocols’ operation. The authors assumed that some random genesis blocks were available from the initial trusted setup. They also stated that Markov models needed to be extremely complex in order to capture the dynamics of such complicated systems.
[57]	*Bitcoin:* Selfish mining attack.	MDP	Profit threshold for selfish mining attacks aimed at capturing different blockchain instances, which have various stale block rates and confirmations. The authors stressed that a malicious user in this system would eventually succeed in case he/she executed double-spending simultaneously.
[58]	*Bitcoin, Litecoin, and Dogecoin:* Selfish mining attack.	MDP	The authors proposed a flexible model with flexible integration of different PoW blockchain instances. It was supported by a standalone security abstraction layer. The systems allowed executing various attacks, corresponding optimal execution strategies, and the impact on the operation.
[59]	*Bitcoin:* Selfish mining attack defense strategy.	MDP	The authors analyzed the current fork resolution issue and highlighted the need for its revision by introducing the fork-resolving policy based on weights.
[65]	*Bitcoin:* Double spending attack.	RW	The author proposed to analyze probabilistically the executability of the attack. The implementing block confirmation mechanism was considered as a significant potential improvement [67].
[66]	*Bitcoin:* Double spending attack.	RW	The authors proposed the analysis of the different mining strategies’ profitabilities, in particular with the honest strategy. It was mentioned that difficulty adjustment during the attack should be considered in more detail when similar techniques are developed.

**Table 3:** Main blockchain emulation tools: PU, Public Blockchain; PR, Private Blockchain.

#	Tool	Type	Scalability	Main Application	Main Challenge
[28]	Blockbench	PU/PR	Low	First academia-driven framework allowing analyzing different blockchains, e.g, Ethereum, Parity, and Hyperledger Fabric.	The project is inactive.
[62]	BlockLite	PU	Low	An academia-driven work aimed to allow the emulation of the blockchain on a single node.	The project is inactive.
[79]	Corda Testing Tools	PR	High	Enterprise solution allowing evaluating Corda operation, as well as new DApps before the actual integration.	Limited to R3 Corda.
[74]	Ethereum Tester	PR	n/a	Ethereum-driven open-source library allowing testing DApps and smart contracts.	Designed specifically for Ethereum developers and lacks community support.
[80]	Exonum Testkit	PR	High	Private (enterprise) Exonum blockchain emulator.	Limited to Exonum.
[69]	Hyperledger Tools	PR	High	Community-driven private blockchain performance analysis toolbox supporting high tps and various implementations.	Extreme complexity of the development environment.
[76]	Truffle Suite	PR	High	Allows flexible development of Ethereum-based DApps and smart contracts’ operation with strong community support.	n/a
[71]	VIBES	PU	Medium	An academia-driven emulator developed for Bitcoin system analysis. An excellent educational example with detailed explanations of the system operation.	The project is inactive.
